# Supplementary material for: Sensory Processing Sensitivity and Gastrointestinal Symptoms in Japanese Adults
Source: Int J Environ Res Public Health. 2022 Aug 11;19(16):9893. doi: 10.3390/ijerph19169893 (PMC9408471; doi:10.3390/ijerph19169893)
Supplement: Supplementary file 1 [file ijerph-19-09893-s001.zip › ijerph-1841906-supplementary.pdf]

Supplementary Materials

Sensory Processing Sensitivity and Gastrointestinal Symptoms in Japanese Adults

Shuhei Iimura and Satoshi Takasugi

----- Table of Contents -----

Figure S1: *Histogram of Sensory Processing Sensitivity*

Figure S2: *Histogram of Gastrointestinal Disease Symptoms*

Table S1: *Descriptive Statistics (Gender)*

Table S2: *Descriptive Statistics (Major and Professional Experience in Nutrition and Health)*

Table S3: *Descriptive Statistics (Number of Family Members Living Together)*

Table S4: *Descriptive Statistics (Marital Status)*

Table S5: *Descriptive Statistics (Number of Children Living Together)*

Table S6: *Descriptive Statistics (Annual Household Income)*

Table S7: *Descriptive Statistics (Frequency of Alcohol Consumption)*

Table S8: *Descriptive Statistics (Smoking Habit)*

Table S9: *Descriptive Statistics (Dietary Advice and Therapy)*

Table S10: *Descriptive Statistics (Physical Activity Level)*

Table S11: *Descriptive Statistics (Education)*

Table S12: *Descriptive Statistics (Food Allergy)*

Table S13: *Zero-Order Correlations between Variables*

Figure S1

Histogram of Sensory Processing Sensitivity

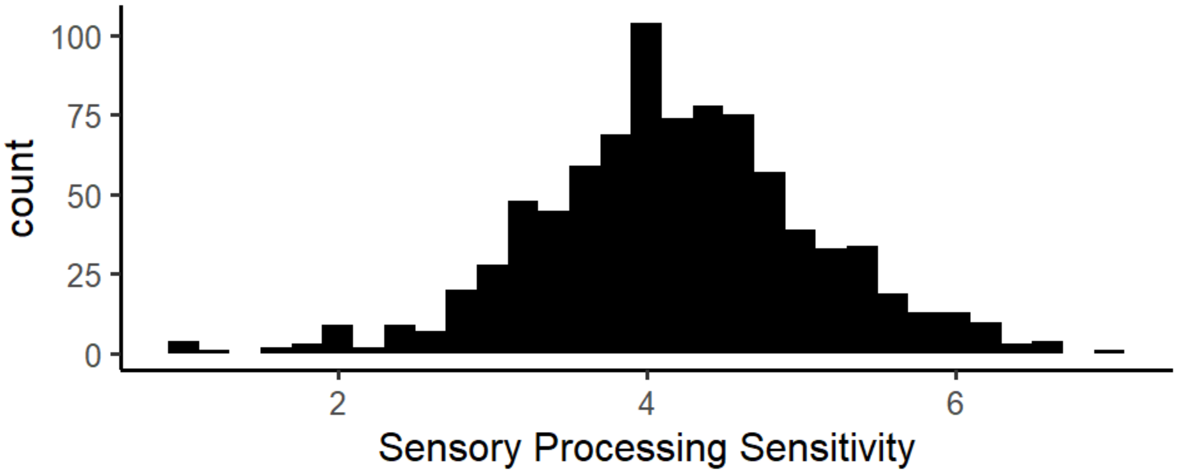

Figure S2

Histogram of Gastrointestinal Disease Symptoms

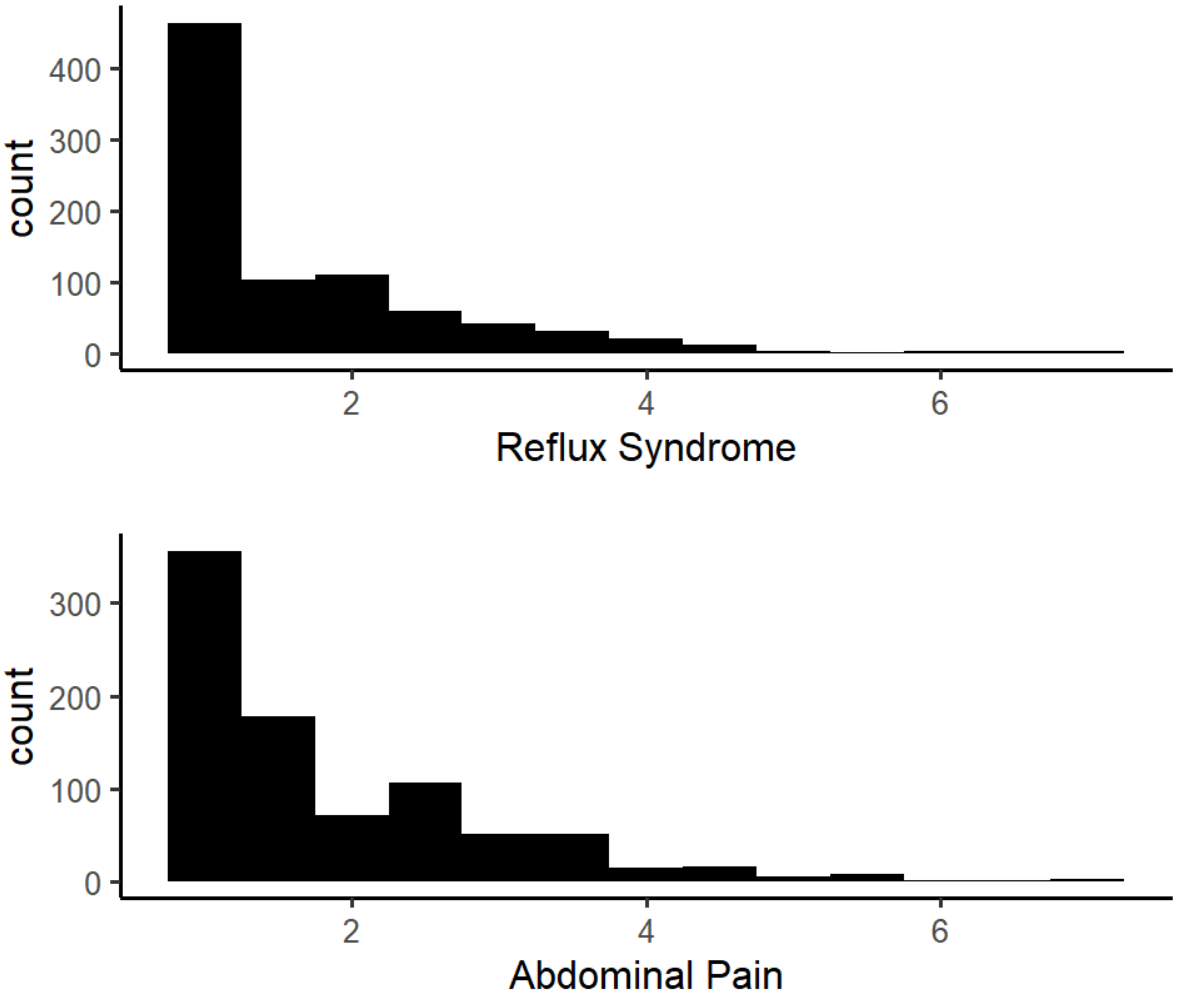

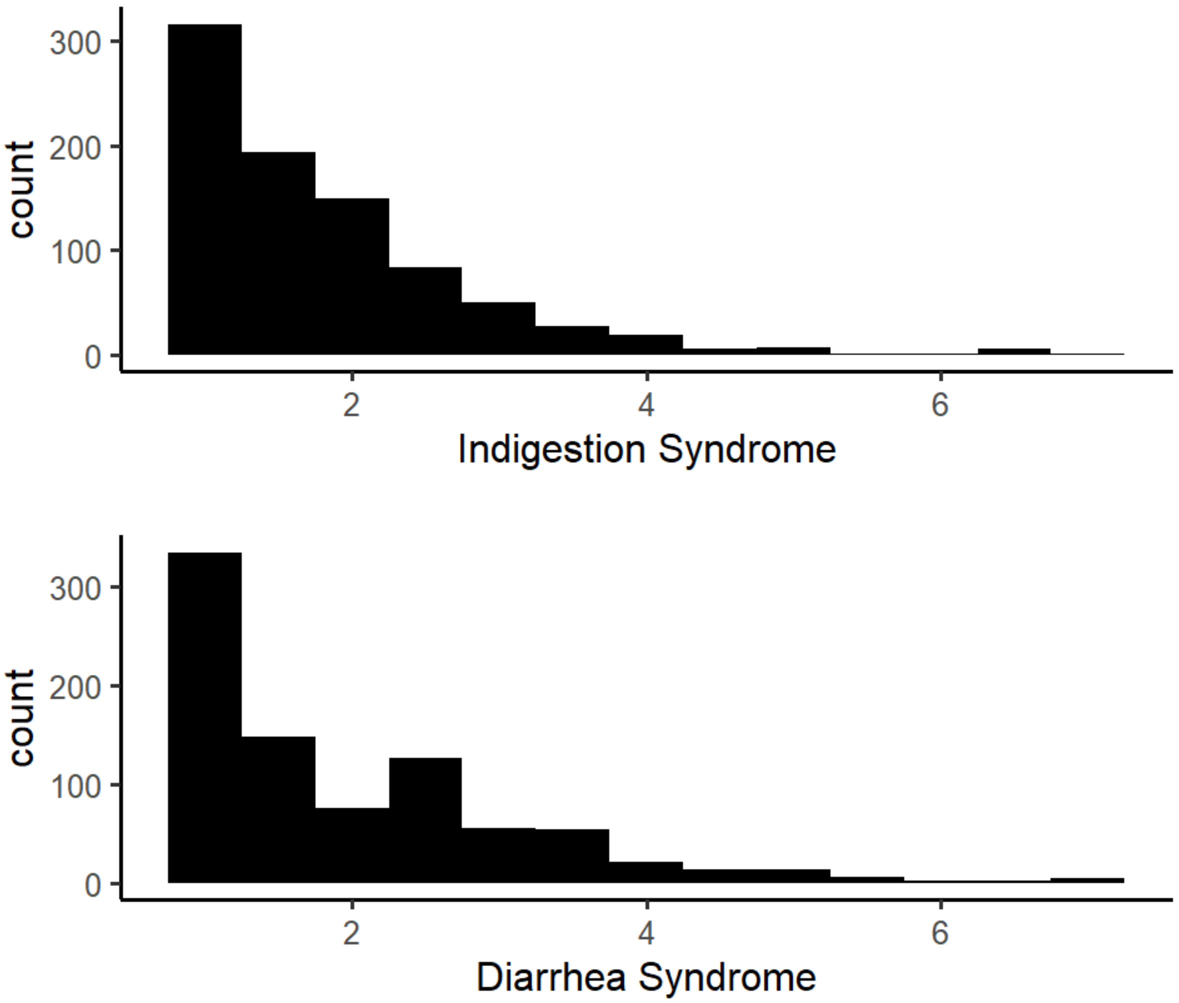

count
300
200
100
0
2
4
6
Indigestion Syndrome
count
300
200
100
0
2
4
6
Diarrhea Syndrome

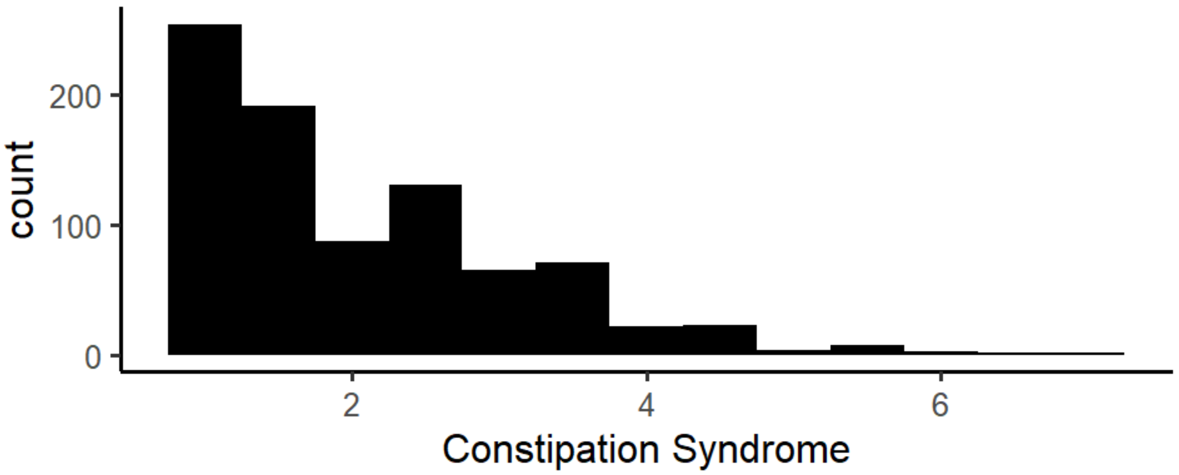
200
100
0
count
2
4
6
Constipation Syndrome

Table S1

Descriptive Statistics (Gender)

	Female		Male		$t(df = 861)$	d	p
	M	SD	M	SD			
Sensory processing sensitivity	4.31	0.90	4.05	0.90	4.20	0.29	<.001
Reflux symptoms	1.76	1.08	1.63	1.02	1.93	0.13	.053
Abdominal pain	2.02	1.15	1.67	0.96	4.74	0.32	<.001
Indigestion symptoms	2.02	1.03	1.76	0.93	3.85	0.26	<.001
Diarrhea symptoms	2.02	1.17	1.93	1.17	1.12	0.08	.265
Constipation symptoms	2.37	1.15	1.75	0.98	8.48	0.58	<.001

Table S2

Descriptive Statistics (Major and Professional Experience in Nutrition and Health)

	Yes		No		$t(df = 861)$	d	p
	M	SD	M	SD			
Sensory processing sensitivity	4.19	0.96	4.18	0.90	0.04	0.00	.965
Reflux symptoms	1.92	1.20	1.66	1.02	2.39	0.24	.017
Abdominal pain	2.10	1.19	1.81	1.05	2.61	0.26	.009
Indigestion symptoms	2.07	1.06	1.87	0.98	1.94	0.19	.053
Diarrhea symptoms	2.04	1.10	1.97	1.18	0.59	0.06	.558
Constipation symptoms	2.38	1.28	2.02	1.08	3.23	0.32	.001

Table S3

Descriptive Statistics (Number of Family Members Living Together)

	One		Two		Three		Four		Five		Six		> Seven		F (6, 856)	p	post hoc[a]
	M	*SD*	*M*	*SD*	*M*	*SD*	*M*	*SD*	*M*	*SD*	*M*	*SD*	*M*	*SD*			
Sensory processing sensitivity	4.22	0.90	4.24	0.94	4.14	0.91	4.22	0.85	4.13	0.86	4.28	0.83	3.48	1.20	1.76	.104	NA
Reflux symptoms	1.70	1.04	1.83	1.10	1.64	1.09	1.63	0.98	1.64	1.07	1.91	1.00	1.93	0.81	0.95	.457	NA
Abdominal pain	1.77	0.96	2.02	1.15	1.83	1.09	1.81	1.08	1.85	1.20	1.92	1.00	1.88	0.87	0.99	.431	NA
Indigestion symptoms	1.94	0.98	2.01	1.03	1.92	1.08	1.78	0.85	1.76	0.94	1.89	1.00	1.68	0.76	1.21	.300	NA
Diarrhea symptoms	1.97	1.18	2.12	1.19	1.88	1.10	1.90	1.15	2.10	1.28	1.88	1.25	2.52	1.35	1.46	.189	NA
Constipation symptoms	1.99	1.04	2.22	1.12	2.04	1.17	1.97	1.00	2.33	1.32	2.13	1.40	1.90	1.03	1.57	.151	NA

Note. [a] Holm method

Table S4

Descriptive Statistics (Marriage Status)

	Married		Other		$t(df = 861)$	d	p
	M	SD	M	SD			
Sensory processing sensitivity	4.00	0.88	4.31	0.91	4.97	0.34	<.001
Reflux symptoms	1.76	1.08	1.66	1.03	1.47	0.10	.142
Abdominal pain	1.91	1.06	1.81	1.09	1.40	0.10	.162
Indigestion symptoms	1.87	0.88	1.92	1.05	0.67	0.05	.502
Diarrhea symptoms	1.96	1.11	1.98	1.21	0.26	0.02	.793
Constipation symptoms	2.13	1.08	2.03	1.14	1.24	0.09	.214

Table S5

Descriptive Statistics (Number of Children Living Together)

	None		One		Two		Three		> Four		*F* (4, 858)	*p*	post hoc[a]
	M	*SD*	*M*	*SD*	*M*	*SD*	*M*	*SD*	*M*	*SD*			
Sensory processing sensitivity	4.28	0.92	3.90	0.86	4.09	0.86	3.95	0.84	4.18	0.75	5.90	<.001	One > None
Reflux symptoms	1.69	1.06	1.60	1.06	1.84	1.07	1.69	0.85	2.17	0.88	1.03	.393	NA
Abdominal pain	1.87	1.10	1.69	0.88	2.03	1.21	1.76	0.98	1.94	0.90	1.53	.192	NA
Indigestion symptoms	1.93	1.04	1.82	0.90	1.93	0.88	1.61	0.61	1.67	0.82	1.12	.344	NA
Diarrhea symptoms	2.00	1.20	1.75	1.00	2.08	1.18	2.15	1.18	1.94	1.06	1.73	.142	NA
Constipation symptoms	2.08	1.13	1.87	1.05	2.27	1.08	2.27	1.08	2.06	1.00	2.12	.076	NA

Note. [a] Holm method

Table S6

Descriptive Statistics (Annual Household Income)

	[1] < 2,000,000 yen		[2] 2,000,000 ~ 4,000,000 yen		[3] 4,000,000 ~ 6,000,000 yen		[4] 6,000,000 ~ 8,000,000 yen		[5] 8,000,000 ~ 10,000,000 yen		[6] 10,000,000 ~ 12,000,000 yen		[7] > 12,000,000 yen		*F* (6, 856)	*p*	post hoc[a]
	M	*SD*	*M*	*SD*	*M*	*SD*	*M*	*SD*	*M*	*SD*	*M*	*SD*	*M*	*SD*			
Sensory processing sensitivity	4.58	0.92	4.18	0.87	4.12	0.88	4.15	0.90	4.04	0.92	4.27	0.73	4.10	1.15	3.35	.003	1 > 2, 3, 4, 5, 7
Reflux symptoms	1.80	1.17	1.72	1.06	1.72	1.05	1.65	0.93	1.61	1.09	1.58	0.90	1.66	1.25	0.40	.880	NA
Abdominal pain	1.98	1.22	1.97	1.14	1.77	0.95	1.86	1.07	1.67	0.90	1.76	0.90	1.82	1.36	1.30	.255	NA
Indigestion symptoms	2.03	1.11	1.91	1.01	1.92	0.93	1.92	0.98	1.77	0.99	1.74	0.90	1.76	1.07	0.81	.564	NA
Diarrhea symptoms	2.16	1.41	2.03	1.18	1.99	1.18	1.89	1.05	1.80	1.02	1.77	0.93	1.97	1.27	1.01	.414	NA
Constipation symptoms	2.29	1.31	2.09	1.10	2.11	1.13	2.05	1.03	1.90	0.97	1.84	1.02	1.89	1.23	1.40	.213	NA

Note. [a] Holm method

Table S7

Descriptive Statistics (Frequency of Alcohol Consumption)

	[1] None		[2] 1 ~ 3 days/month		[3] 1 ~ 2 days/week		[4] 3 ~ 4 days/week		[5] 5 ~ 7 days/week		*F* (4, 858)	*p*	post hoc[a]
	M	*SD*	*M*	*SD*	*M*	*SD*	*M*	*SD*	*M*	*SD*			
Sensory processing sensitivity	4.27	0.91	4.06	0.81	4.20	0.80	3.85	1.02	4.11	1.03	3.80	.005	1 > 4
Reflux symptoms	1.63	1.01	1.74	1.11	1.84	1.09	1.69	0.94	1.74	1.15	1.22	.301	NA
Abdominal pain	1.80	1.06	1.94	1.02	1.96	1.10	1.79	0.98	1.86	1.25	0.92	.450	NA
Indigestion symptoms	1.84	0.92	2.01	1.00	1.92	1.06	1.89	0.87	1.98	1.22	1.01	.399	NA
Diarrhea symptoms	1.93	1.09	1.98	1.09	2.04	1.27	1.93	1.11	2.14	1.52	0.71	.588	NA
Constipation symptoms	2.08	1.12	2.12	1.07	2.04	1.10	1.90	0.90	2.09	1.33	0.46	.763	NA

Note. [a] Holm method

Table S8

Descriptive Statistics (Smoking Habit)

	[1] None		[2] Used to smoke		[3] Currently smoking		*F* (2, 860)	*p*	post hoc[a]
	M	*SD*	*M*	*SD*	*M*	*SD*			
Sensory processing sensitivity	4.25	0.87	4.15	0.96	3.91	0.97	9.24	<.001	1 > 3
Reflux symptoms	1.63	0.97	1.92	1.29	1.86	1.21	4.56	.011	*ns*
Abdominal pain	1.80	1.01	2.03	1.29	1.99	1.20	3.17	.043	*ns*
Indigestion symptoms	1.87	0.92	2.00	1.15	1.98	1.15	1.17	.312	NA
Diarrhea symptoms	1.90	1.09	2.10	1.26	2.22	1.39	5.26	.005	1 > 3
Constipation symptoms	2.07	1.09	1.99	1.04	2.09	1.25	0.21	.812	NA

Note. [a] Holm method

Table S9

Descriptive Statistics (Dietary Advice and Therapy)

	[1] Currently receiving dietary advice and/or therapy		[2] Used to receive dietary advice and/or therapy		[3] No dietary advice and/or therapy received		*F* (2, 860)	*p*	post hoc[a]
	M	*SD*	*M*	*SD*	*M*	*SD*			
Sensory processing sensitivity	4.13	1.26	4.89	0.96	4.17	0.89	5.39	.005	2 > 1, 3
Reflux symptoms	2.16	1.68	2.65	1.50	1.67	1.02	8.89	<.001	2 > 3
Abdominal pain	2.38	1.77	2.73	1.41	1.82	1.04	7.90	<.001	2 > 3
Indigestion symptoms	2.16	1.39	2.53	1.57	1.88	0.96	4.17	.016	2 > 3
Diarrhea symptoms	2.15	1.63	2.43	1.47	1.96	1.15	1.52	.220	NA
Constipation symptoms	2.15	1.51	2.84	1.67	2.05	1.09	4.25	.015	2 > 3

Note. [a] Holm method

Table S10

Descriptive Statistics (Physical Activity Level)

	[1] High		[2] Moderate		[3] Low		*F* (2, 860)	*p*	post hoc[a]
	M	*SD*	*M*	*SD*	*M*	*SD*			
Sensory processing sensitivity	4.06	1.00	4.13	0.90	4.35	0.86	6.31	.002	3 > 1, 2
Reflux symptoms	1.72	1.10	1.70	1.02	1.69	1.10	0.02	.978	NA
Abdominal pain	1.94	1.12	1.81	1.01	1.90	1.19	1.07	.345	NA
Indigestion symptoms	1.90	1.04	1.87	0.94	1.96	1.06	0.69	.503	NA
Diarrhea symptoms	2.23	1.36	1.90	1.05	2.00	1.27	3.88	.021	1 > 2
Constipation symptoms	1.97	1.12	2.00	1.05	2.26	1.21	5.16	.006	3 > 1, 2

Note. [a] Holm method

Table S11

Descriptive Statistics (Education)

	[1] Elementary and middle schools		[2] High school		[3] Junior college		[4] University		[5] Graduate school		F (4, 858)	p	post hoc[a]
	M	*SD*	*M*	*SD*	*M*	*SD*	*M*	*SD*	*M*	*SD*			
Sensory processing sensitivity	4.53	0.88	4.21	0.97	4.21	0.85	4.15	0.89	3.93	0.87	1.78	.130	NA
Reflux symptoms	1.60	0.84	1.72	1.09	1.76	1.21	1.67	0.99	1.63	0.90	0.35	.844	NA
Abdominal pain	1.92	1.14	1.90	1.13	1.88	1.12	1.83	1.02	1.58	1.02	0.71	.583	NA
Indigestion symptoms	1.78	0.87	1.86	0.98	1.94	1.06	1.92	0.98	1.73	0.79	0.55	.696	NA
Diarrhea symptoms	2.17	1.30	2.10	1.31	1.91	1.07	1.94	1.12	1.61	0.82	1.91	.107	NA
Constipation symptoms	2.16	1.05	2.16	1.15	2.14	1.19	2.02	1.08	1.67	0.86	1.87	.113	NA

Note. [a] Holm method

Table S12

Descriptive Statistics (Food Allergy)

	Yes		No		$t(df = 861)$	d	p
	M	SD	M	SD			
Sensory processing sensitivity	4.45	0.80	4.17	0.91	2.15	0.31	.032
Reflux symptoms	2.08	1.41	1.67	1.02	2.67	0.39	.008
Abdominal pain	2.31	1.30	1.82	1.05	3.18	0.46	.002
Indigestion symptoms	2.22	1.29	1.88	0.96	2.37	0.34	.018
Diarrhea symptoms	2.30	1.24	1.95	1.16	2.05	0.30	.040
Constipation symptoms	2.19	1.14	2.06	1.11	0.79	0.11	.431

Table S13

Zero-Order Correlations between Variables

	1	2	3	4	5	6	7	8	9	10	11	12	13	14	15	16	17	18	19
1. Gender	—																		
2. Age	.00	—																	
3. Specialty[1]	-.16**	.06	—																
4. Family[2]	.01	.02	.03	—															
5. Marriage[3]	-.20**	-.27**	.08*	-.24**	—														
6. Children[4]	.18**	.25**	-.02	.50**	-.59**	—													
7. Income[5]	-.03	-.03	-.06	.30**	-.15**	.02	—												
8. Alcohol consumption[6]	.20**	-.10**	-.02	.07	.00	-.01	-.08*	—											
9. Smoking[7]	.22**	-.10**	-.05	-.05	.03	-.08*	.02	.30**	—										
10. Advice/therapy[8]	.04	-.01	.05	-.01	-.05	.03	-.04	.00	.09*	—									
11. Activity[9]	.22**	-.01	.02	-.02	-.05	.01	-.02	.08*	.16**	-.05	—								
12. Education	-.11**	.03	-.10**	-.18**	.01	-.09**	.21**	-.09*	.11**	-.01	-.05	—							
13. Allergy[10]	-.05	.00	.00	.00	.04	-.01	.06	-.03	.06	.03	.02	.04	—						
14. BMI[11]	-.19**	.12**	.01	.03	-.01	-.02	.00	-.01	-.04	-.03	-.01	.02	-.02	—					
15. Sensitivity[12]	.14**	-.02	.00	-.05	.17**	-.12**	-.08*	.10**	.16**	-.04	.11**	-.07*	-.07*	-.06	—				
16. Reflux symptoms	.07	-.01	-.08*	-.02	-.05	.03	-.05	-.05	-.08*	-.11**	-.01	-.02	-.09**	.06	.19**	—			
17. Abdominal symptoms	.16**	-.05	-.09**	.00	-.05	.01	-.06	-.02	-.06	-.11**	.00	-.04	-.11**	.01	.24**	.76**	—		
18. Indigestion symptoms	.13**	-.05	-.07	-.07*	.02	-.05	-.06	-.04	-.04	-.07*	.03	.02	-.08*	.03	.25**	.63**	.71**	—	
19. Diarrhea symptoms	.04	-.04	-.02	.01	.01	.00	-.07	-.05	-.10**	-.04	-.04	-.08*	-.07*	.07*	.20**	.56**	.62**	.65**	—
20. Constipation symptoms	.28**	-.04	-.11**	.01	-.04	.03	-.09**	.01	.01	-.05	.10**	-.08*	-.03	-.06	.26**	.47**	.53**	.60**	.62**

[1]Specialty = Major and professional experience in nutrition and health. [2]Family = Number of family members living together. [3]Marriage = marital status. [4]Children = Number of children living together. [5]Income = annual household income. [6]Alcohol consumption = Frequency of alcohol consumption. [7]Smoking = smoking habit. [8]Advice/therapy = Dietary advice and therapy. [9]Activity = Physical activity level. [10]Allergy = Food

allergy. [11]BMI = Body Mass Index. [12]Sensitivity = sensory processing sensitivity. N = 863.

* p <.05, ** p <.01, *** p <.001
